# The Potential Use of Salivary miRNAs as Promising Biomarkers for Detection of Cancer: A Meta-Analysis

**DOI:** 10.1371/journal.pone.0166303

**Published:** 2016-11-10

**Authors:** Yuanjie Ding, Qing Ma, Fen Liu, Lei Zhao, Wenqiang Wei

**Affiliations:** 1 Department of Epidemiology and Health Statistics, School of Public Health, Beijing Municipal Key Laboratory of Clinical Epidemiology, Capital Medical University, Beijing, China; 2 Department of Cancer Epidemiology, National Cancer Center/Cancer Hospital, Chinese Academy of Medical Science & Peking Union Medical College, Beijing, China; 3 Department of Molecular Physiology and Biophysics, Holden Comprehensive Cancer Center, University of Iowa Carver College of Medicine, Iowa City, IA, United States of America; The Ohio State University, UNITED STATES

## Abstract

**Background:**

Accumulating evidence has demonstrated that microRNAs (miRNAs) could serve as promising molecular biomarkers for cancer detection. This study aims to systematically assess the diagnostic performance of salivary miRNAs in detection of cancer through a comprehensive meta-analysis.

**Methods:**

Eligible studies were identified using PubMed and other computerized databases up to October 31, 2015, supplemented by a manual search of references from retrieved articles. The pooled sensitivity, specificity, and other measurements of accuracy of salivary miRNAs in the diagnosis of cancer were analyzed using the bivariate binomial mixed model.

**Results:**

Seventeen studies from 8 articles with 694 subjects were included in this meta-analysis. All studies have a relatively high score of quality assessment. The overall sensitivity, specificity, positive likelihood ratio (PLR), negative likelihood ratio (NLR), and diagnostic odds ratio (DOR) of salivary miRNAs in detection of cancer were 0.77 (95% confidence intervals [CI]: 0.69–0.84), 0.77 (95%CI: 0.65–0.88), 3.37 (95%CI: 2.26–5.02), 0.29 (95%CI: 0.23–0.38), and 11.41 (95%CI: 7.35–17.73), respectively. The AUC was 0.84 (95%CI: 0.80–0.87). Moreover, both whole saliva and saliva supernatant could be used as sources of clinical specimens for miRNAs detection.

**Conclusions:**

Our meta-analysis demonstrated that salivary miRNAs may serve as potential noninvasive biomarkers for cancer detection. The findings need to be confirmed with further research before it can be applied in the clinic.

## Introduction

According to the report of Globocan 2012 published by the International Agency for Cancer Research, there has been an estimated figure of 14.1 million cancer cases and 8.2 million cancer-related deaths worldwide in 2012 [1]. In China, cancer is one of the major chronic diseases that adversely affect the health status of individuals, being ranked the first and the second in causes of death for people living in urban and rural areas, respectively [2]. Early detection is critical in improving the prognosis of cancer patients. For example, the overall 5-year survival rate for esophageal squamous cell carcinoma (ESCC) is low, ranging from 3% to 5% [3]. This rate, however, could be increased to 90% if tumors were diagnosed and treated at an early stage [4]. The current techniques for early detection of cancer, such as endoscopy and serum tumor markers, are generally invasive or lack sufficient sensitivity and specificity, which makes them hard to be performed in a large population [5]. Therefore, novel noninvasive biomarkers for early detection of cancers are urgently needed.

MicroRNAs (miRNAs) are a class of small noncoding RNAs of about 18–25 nucleotides in length, which are highly conserved during evolution. These miRNAs post-transcriptionally regulate gene expression by binding to 3’-untranslated region (3’-UTR) of target messenger RNAs according to base pair complementarity. This results in RNA degradation and/or translational inhibition [6]. The aberrations in miRNA expression have been reported to be involved in tumorigenesis and cancer development [7]. The expression patterns of miRNAs are cell- or tissue-specific, which may be helpful in early diagnosis of different types of cancers and in predicting survival and prognosis of patients. Previous studies have demonstrated that miRNAs exist in various body fluids, including saliva, and the expression levels of salivary miRNAs were stable and did not change with time in the same person [8–10]. As saliva collection is simple, noninvasive, and easily accessible, salivary miRNAs show potential values in early detection of diseases, including cancer. Several groups have reported the potential use of salivary miRNAs as promising biomarkers for detection of cancer, particularly digestive tract cancers. However, these studies have reported inconsistent results. Wu *et al*. [11] found that miR-144 was highly expressed in both whole saliva and saliva supernatant of patients with esophageal cancer and can be considered as a novel biomarker for esophageal cancer detection. In the whole saliva, the sensitivity was 74.6% and the specificity was 92.0%; in saliva supernatant, the sensitivity and specificity was 53.7% and 94.0%, respectively. Ye *et al*. [12] reported that salivary miRNA-21 yields a diagnostic characteristic with a sensitivity of 89% and a specificity of 64% for detection of early esophageal cancer. Therefore we conducted a systematic and comprehensive meta-analysis of all eligible studies to explore the overall diagnostic values of salivary miRNAs as promising biomarkers for cancer detection.

## Materials and Methods

### Search strategy and study selection

A comprehensive literature search was carried out in the PubMed, Embase, the Cochrane Library, ISI Web of Science databases, Chinese Wanfang database and China National knowledge Infrastructure (CNKI) up to October 31, 2015 without language limitation. The key words used for literature retrieval were as follows: (“Saliva” OR “Spit” OR “Spittle”) AND (“MicroRNAs” OR “miRNAs” OR “miR”) AND (“Cancer” OR “Carcinoma” OR “Tumor”). This study was performed in accordance with the PRISMA statement checklist (S1 PRISMA checklist).

**Fig 1:**
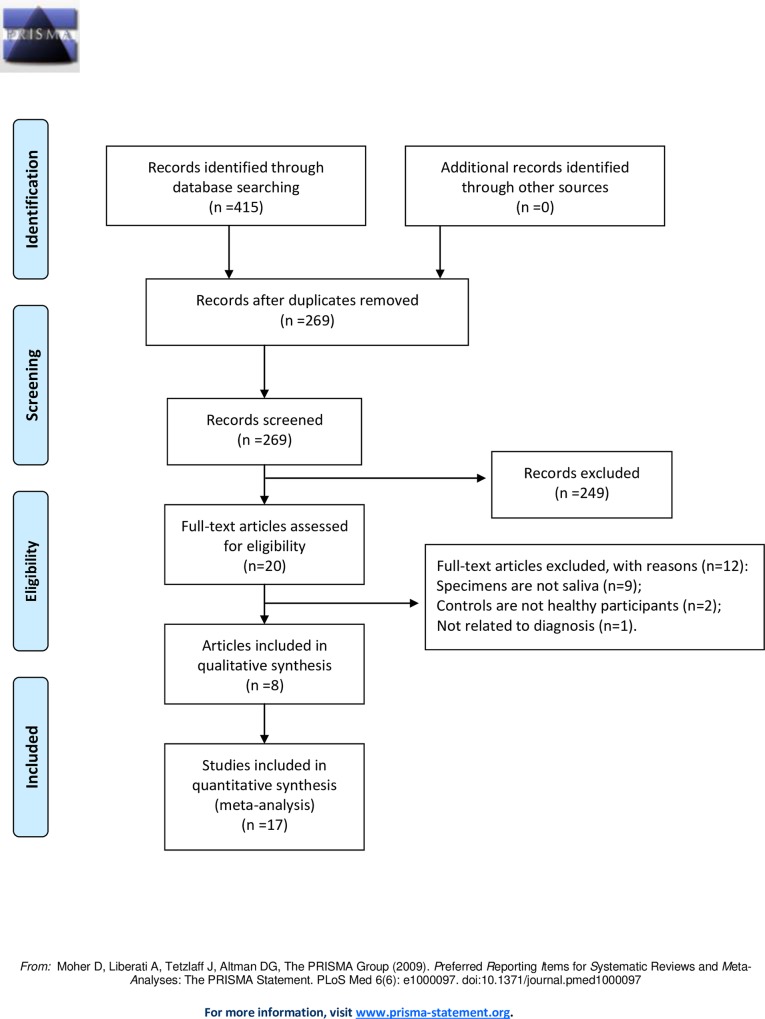
PRISMA 2009 Flow Diagram in our study.

A study was considered eligible only if the publication met all of the following criteria: (1) the study was a diagnostic study using salivary miRNAs; (2) subjects included cancer patients and healthy controls; (3) sufficient data was available for generating two-by-two tables which consist of true positive (TP), false positive (FP), true negative (TN) and false negative (FN). Furthermore, we manually examined the bibliographies in these studies and related reviews to identify any potentially eligible study. The process was performed independently by two reviewers. The reasons for excluding studies are described in Fig 1 which followed the format of PRISMA 2009 Flow Diagram.

### Data extraction

Estimates of TP, FP, TN and FN were extracted from eligible studies independently by two investigators. In addition, basic characteristics of studies, including the first author’s last name, year of publication, country of study, ethnicity, sample sizes, miRNAs expression signature, cancer type and specimen origin were recorded.

### Quality assessment

The quality of the included studies was evaluated independently by two investigators using the revised Quality Assessment of Diagnostic Accuracy Studies (QUADAS-2) [13]. The QUADAS-2 is a revised tool for the qualitative assessment of diagnostic accuracy studies, which comprises four key domains: patient selection, index test, reference standard, and flow and timing.

### Statistical analyses

Data analyses were performed using Meta-DiSc statistical software version 1.4 and Stata statistical software (v.13.0; Stata Corp, College Station, TX, USA). The Spearman correlation coefficient of logarithm sensitivity and 1-specificity was calculated to detect the threshold effect [14]. A χ2-based Cochran's Q test and Higgins’ *I*^*2*^ statistics were used to assess the heterogeneity among studies. A value of *P*<0.1 and *I*^*2*^>50% was considered significant heterogeneity [15]. The bivariate binomial mixed model was employed to summarize the pooled sensitivity, specificity, positive likelihood ratio(PLR), negative likelihood ratio(NLR) and diagnostic odds ratio (DOR) with 95% confidence intervals (CIs) [16]. A summary receiver operating characteristic curve (SROC) was constructed based on pooled sensitivity and specificity of included studies [17]. The area under the SROC curve (AUC) represents an analytical summary of test performance [18]. In addition, subgroup analyses and meta-regression were performed to explore the potential heterogeneity among included studies. Finally, publication bias was investigated by using Deek’s funnel plot [19]. All *P* values were two sided.

## Results

### Characteristics of included studies and quality assessment

The literature search results of this meta-analysis are presented in Fig 1. Four hundreds and fifteen records were identified through systematic search and manual review for initial search, and 269 abstracts were remained after removing duplicate records. After titles and abstracts were reviewed, 20 articles of the non-duplicate records were subjected to further full-text review, of which 12 were excluded according to the exclusion criteria. Finally, 8 articles were included in the present meta-analysis (Table 1) [11, 12, 20–25].

**Table 1:** Main characteristics of the eight studies included in this meta-analysis.

Included studies	Year	Country	Ethnicity	Case/control	miRNAs	Cancer spectrum	Sample	QUADAS-2
Xie *et al*.	2012	China	Asian	32/16	miR-21(up-regulated)	ESCC	Saliva supernatant	5
Wu e*t* al.	2013	China	Asian	67/50	miR-144(up-regulated)	Esophageal cancer	Whole salivaSaliva supernatant	5
Xie *et al*.	2013	China	Asian	39/19	miR-10b*、21、144、451(up-regulated)	Esophageal cancer	Whole salivaSaliva supernatant	5
Ye *et al*.	2014	China	Asian	100/50	miR-21(up-regulated)	ESCC	Saliva supernatant	5
Xie *et al*.	2014	China	Asian	40/40	miR-940(up-regulated)、3679-5p(down-regulated)	Pancreatic cancer	Saliva supernatant	5
Momen-Heravi *et al*.	2014	America	White/Non-white	9/9	miR-27b(up-regulated)、136(down-regulated)	OSCC	Saliva supernatant	5
Li *et al*.	2015	China	Asian	112/100	miR-21(up-regulated)	Esophageal cancer	Saliva supernatant	5
Humeau *et al*.	2015	France	Not mentioned	7/4	miR-21、23a、23b、29c(up-regulated)	Pancreatic cancer	Saliva supernatant	5

ESCC, esophageal squamous cell carcinoma; OSCC, oral squamous cell carcinoma; QUADAS-2, the revised Quality Assessment of Diagnostic Accuracy Studies.

In total, 17 studies from 8 included articles covering 11 types of miRNAs and 694 subjects (406 patients with cancers and 288 controls) were available in this meta-analysis. The articles of Xie *et al*.(2012), Wu *et al*. (2013), Ye *et al*. (2014) and Li *et al*. (2015) provided a single study. The publications of Xie *et al*. (2013), Xie *et al*. (2014), Momen-Heravi *et al*. (2014) and Humeau *et al*. (2015) included 4, 3, 2 and 4 studies, respectively. The sample type of all 8 articles were saliva supernatant. The publication years of the included articles were from 2012 to 2015. The patient spectrum was composed of esophageal squamous cell carcinoma (ESCC), esophageal cancer (EC), pancreatic cancer and oral squamous cell carcinoma (OSCC). Among the 17 studies, 11 studies were conducted in China, 2 in America and 4 in France. Sixteen of these 17 studies investigated the diagnostic value of a single miRNA assay, while only 1 study focused on a panel of 2 miRNAs[21].

The quality of the included articles was assessed by calculating the QUADAS-2 scores (S1 Fig). Overall, all included studies received a score of 5, which indicated a moderately high quality (Table 1).

### Diagnostic accuracy of salivary miRNAs in cancer detection

**Fig 2:**
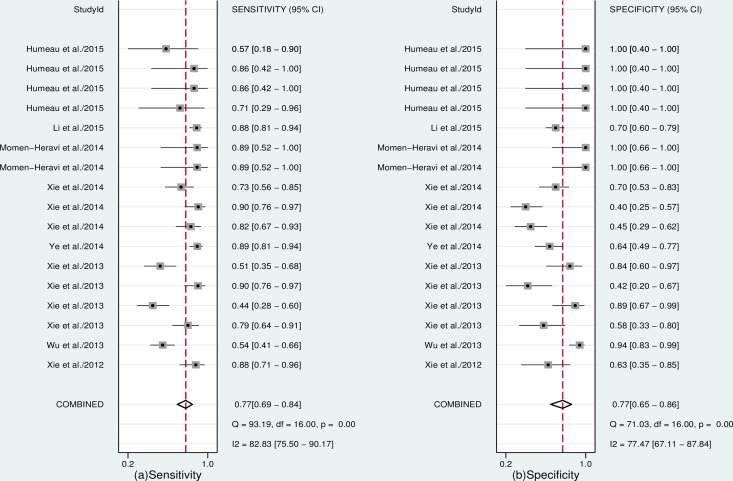
Forest plots of pooled sensitivity (a) and specificity (b) of salivary miRNAs for the diagnosis of cancer.

**Fig 3:**
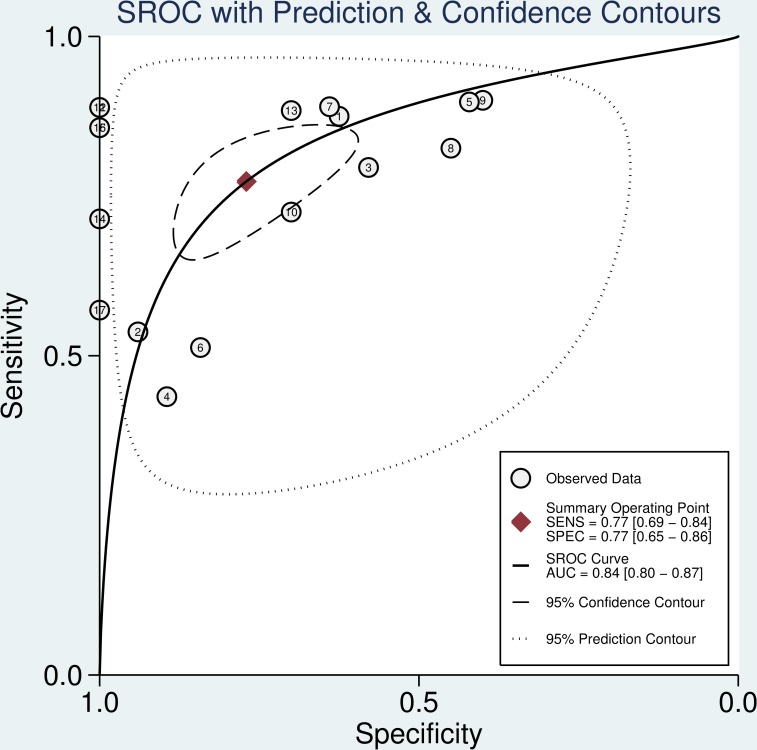
SROC curve with pooled estimates of sensitivity, specificity and AUC on the diagnostic value of salivary miRNAs in cancer.

**Fig 4:**
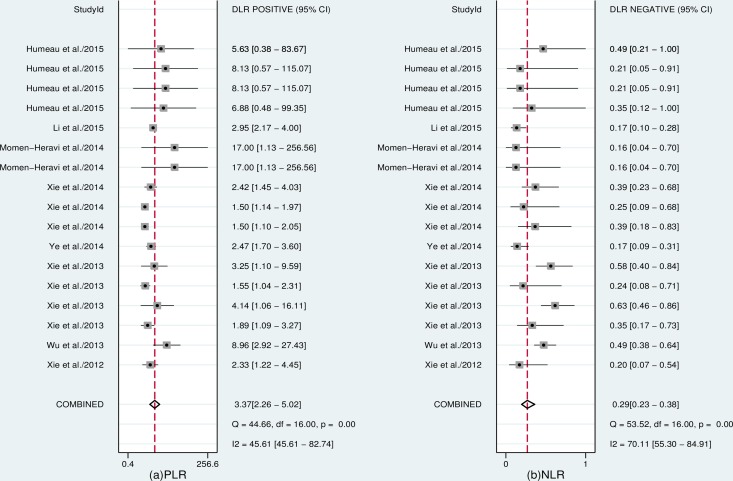
Forest plots of pooled PLR (a) and NLR (b) of salivary miRNAs for the diagnosis of cancer.

**Fig 5:**
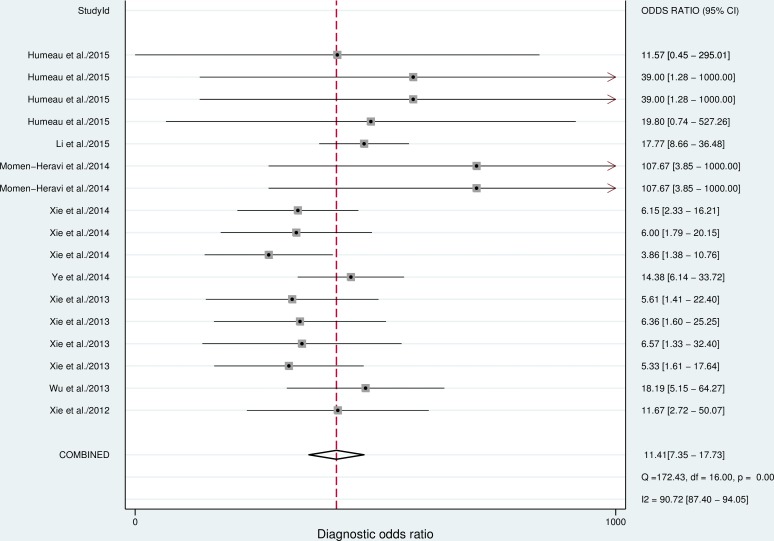
Forest plots of pooled DOR of salivary miRNAs for the diagnosis of cancer.

Seventeen datasets were finally included in this meta-analysis. The estimates of pooled sensitivity, specificity, and AUC were 0.77 (95%CI: 0.69–0.84) (Fig 2A), 0.77 (95%CI: 0.65–0.88) (Fig 2B) and 0.84 (95%CI: 0.80–0.87) (Fig 3), respectively; The overall PLR, NLR and DOR were 3.37 (95%CI: 2.26–5.02) (Fig 4A), 0.29 (95%CI: 0.23–0.38) (Fig 4B) and 11.41 (95%CI: 7.35–17.73) (Fig 5), respectively.

In addition to the sample type of saliva supernatant, there were four studies included (from two articles) investigating esophageal cancer detection using whole saliva as samples [11, 20]. We therefore analyzed the roles of miRNAs isolated from the whole saliva in cancer detection. The pooled sensitivity was 0.86 (95%CI: 0.77–0.92), specificity was 0.68 (95%CI: 0.44–0.85), PLR was 2.69 (95%CI: 1.43–5.07), NLR was 0.21 (95%CI: 0.13–0.31), DOR was 13.11 (95%CI: 5.95–28.92), and the AUC was 0.87 (95%CI: 0.84–0.90).

### Test of heterogeneity and subgroup analysis

In this meta-analysis, the Spearman correlation coefficient was 0.464 with a *P* value of 0.061, suggesting that no obvious heterogeneity was detected as a result of the threshold effect.

We also investigated the non-threshold effect, the results indicated the existence of significant heterogeneity in the overall sensitivity (*I*^*2*^ = 82.83%, *P*<0.001), specificity (*I*^*2*^ = 77.47%, *P*<0.001), PLR (*I*^*2*^ = 45.61%, *P*<0.001), NLR (*I*^*2*^ = 70.11%, *P*<0.001), and DOR (*I*^*2*^ = 90.72%, *P*<0.001). Therefore, a bivariate binomial mixed model was applied to summarize the pooled estimates in this study. To determine the sources of heterogeneity, we performed meta-regression to test the effects of ethnicity (Asian/others), sample size (n≥100/n<100), cancer spectrum (esophageal cancer/others) and miRNA expression patterns (up-regulated/down-regulated) on heterogeneity. The results indicated that sample size was a potential source of heterogeneity in this study (*P* = 0.0205, S1 Table). Consequently, we conducted a subgroup analysis based on sample size (the sample size of 3 studies was greater than 100 and the other 14 studies had a sample size less than 100). As shown in Table 2, the results indicated that as the sample size increased from n<100 to n≥100, the diagnostic accuracy was not significantly elevated. However, the sensitivity increased from 0.75 to 0.80; the specificity increased from 0.64 to 0.75; the PLR increased from 2.06 to 3.13; the NLR decreased from 0.37 to 0.25; and the DOR increased from 6.98 to 16.57.

**Table 2:** Detail information of subgroup analysis.

Analysis	No. of studies	Sensitivity(95%CI)	Specificity(95%CI)	PLR(95%CI)	NLR(95%CI)	DOR(95%CI)	AUC(95%CI)
Ethnicity							
Asian	11	0.77(0.74–0.81)	0.66(0.6q-0.71)	2.20(1.70–2.85)	0.34(0.24–0.48)	8.93(6.28–12.69)	0.83(0.79–0.86)
Others	6	0.86(0.80–0.91)	0.78(0.70–0.84)	3.21(2.39–4.31)	0.23(0.16–0.33)	21.13(11.19–39.91)	0.90(0.87–0.92)
Sample size							
n≥100	3	0.80(0.75–0.85)	0.75(0.68–0.80)	3.13(2.03–4.82)	0.25(0.10–0.62)	16.57(10.01–27.42)	0.87(0.85–0.89)
n<100	14	0.75(0.70–0.80)	0.64(0.58–0.70)	2.06(1.57–2.71)	0.37(0.28–0.49)	6.98(4.61–10.55)	0.81(0.77–0.84)
Cancer spectrum							
Esophageal cancer	8	0.76(0.72–0.80)	0.72(0.67–0.77)	2.51(1.87–3.37)	0.33(0.21–0.52)	11.50(7.78–17.00)	0.84(0.80–0.87)
Others	9	0.81(0.75–0.87)	0.62(0.54–0.70)	2.30(1.43–3.68)	0.33(0.24–0.46)	7.98(4.21–15.14)	0.83(0.79–0.86)
MiRNAs expression							
Up-regulated	14	0.77(0.73–0.81)	0.71(0.66–0.75)	2.56(1.87–3.50)	0.32(0.22–0.45)	11.53(8.04–16.54)	0.85(0.82–0.88)
Down-regulated	2	0.84(0.70–0.93)	0.55(0.40–0.69)	3.82(0.25–58.85)	0.31(0.15–0.67)	13.86(0.56–340.75)	0.81(0.77–0.85)

CI, confidence interval; PLR, positive likelihood ratio; NLR, negative likelihood ratio; DOR, diagnostic odds ratio; AUC, area under the curve.

Although the meta-regression results were negative in the other factors, we still performed subgroup analyses based on these factors to further explore the potential diagnostic value of miRNAs included in this study. The subgroup analysis performed between the ethnicity of subjects (Asian vs. others) indicated that the accuracy of miRNA in Asian patients (sensitivity, 0.77; specificity, 0.66; PLR, 2.20; NLR, 0.34; DOR, 8.93; AUC, 0.83) was not obviously different from patients of other ethnicities (sensitivity, 0.86; specificity, 0.78; PLR, 3.21; NLR, 0.23; DOR, 21.13; AUC, 0.90). We also conducted subgroup analysis based on cancer spectrum, in which eight studies focused on esophageal cancer and nine investigated pancreatic cancer and oral squamous cell carcinoma. There were no significant difference in sensitivity (0.76 vs. 0.81), specificity (0.72 vs. 0.62), PLR (2.51 vs. 2.30), NLR (0.33 vs. 0.33), DOR (11.50 vs. 7.98), and AUC (0.84 vs. 0.83) between the esophageal cancer and the non-esophageal cancer groups. Regarding the expression pattern, sensitivity of up-regulated miRNAs was 0.77, specificity was 0.71, PLR was 2.56 and NLR was 0.32, with a pooled DOR of 11.53 and AUC of 0.85. For the down-regulated miRNAs, sensitivity was 0.84, specificity was 0.55, PLR was 3.82, and NLR was 0.31 with a pooled DOR of 13.86 and AUC of 0.81. No significant difference in these parameters was found between up-regulated miRNAs and down-regulated miRNAs (Table 2).

### Sensitivity analysis and publication bias

Sensitivity analysis was conducted to confirm that our findings were not significantly influenced by any individual study (S2 Fig). The Deek’s funnel plot with the asymmetry test was performed to explore any potential publication bias in this meta-analysis. The slope coefficient reflected a *P*-value of 0.48 in overall studies, suggesting symmetry of the data and the absence of significant publication bias.

## Discussion

Accumulating evidence has demonstrated that miRNAs are stable in body fluids such as saliva and have a great potential to become noninvasive screening tools for cancer detection [8, 9, 26, 27]. To our knowledge, this study is the first systematic review and meta-analysis to evaluate the diagnostic value of salivary miRNAs in discriminating cancer cases. Our meta-analysis included 8 eligible articles (17 studies) with 406 cancer patients and 288 healthy controls. The overall analysis has shown a moderate diagnostic accuracy of salivary miRNAs with an AUC of 0.84, sensitivity of 0.77, and specificity of 0.77. The pooled DOR in our meta-analysis was 11.41, which indicates a moderate level of overall accuracy to discriminate between cancer patients and healthy controls. The overall PLR value was 3.37, suggesting that the probability of having cancer in a person with a positive test result was approximately 3-fold higher compared to healthy controls. The NLR refers to the probability of a cancer patient having a negative test divided by the probability of a person without cancer having a negative test. In the meta-analysis, we found an NLR value of 0.29, indicating that the probability of a patient having cancer is 29% if the miRNA assay shows a negative result. Together, these results revealed that salivary miRNAs have a relatively higher diagnostic efficiency as clinical biomarkers for cancer detection.

In addition to the sample type of saliva supernatant, we also analyzed the roles of salivary miRNAs isolated from the whole saliva in cancer detection. The pooled sensitivity was 0.86, specificity was 0.68, PLR was 2.69, NLR was 0.21, DOR was 13.11, and the AUC was 0.87. The results were consistent between miRNAs from whole saliva and saliva supernatant, suggesting that both types of sample could be used as sources of clinical specimens for miRNAs detection. In support of this notion, Park *et al*. [8] reported that there was similar miRNA expression levels in both whole saliva and saliva supernatant in patients with oral cancer.

Heterogeneity in meta-analysis refers to the variation in study outcomes between studies which might compromise the validity of a systematic review. Therefore, assessment of the consistency of effects across studies is an essential part of a meta-analysis. The threshold effect is one of the primary causes of heterogeneity among diagnostic accuracy studies. In the present meta-analyses, we did not find obvious heterogeneity as a result of the threshold effect. Moreover, we performed meta-regression to test the effect of ethnicity, sample size, cancer type, and expression of miRNAs. The result suggested that sample size might be a source of heterogeneity for this study (*P* = 0.0205, S1 Table). However, the results from subgroup studies suggested that the subgroup of n≥100 showed no statistically significantly higher accuracy than n<100 (Table 2).

We also conducted subgroup analyses based on ethnicity, cancer spectrum and miRNAs expression pattern. The results revealed that no significant differences in diagnostic accuracy were observed between subgroups. However, further studies need to be conducted to confirm these findings. In addition, sensitivity analysis and Deek’s funnel plot asymmetry test were conducted to detect outliers and publication bias and to confirm the robustness of our results.

This meta-analysis was conducted in compliance with the PRISMA guideline, using multiple search strategies and by independent reviewers. We have carefully defined the inclusion and exclusion criteria so that all the studies included in our meta-analysis had acceptable quality and the cases and controls were collated from all included studies. We have used appropriate statistical methods and interpretation through which the statistical power was significantly increased. However, the limitations of this study also need to be addressed. First, the number of involved participants in the eight included articles is quite small, thus larger cohorts are required to confirm the conclusions in further researches. Second, subgroup analysis on an individual miRNA marker could not be conducted due to restricted information and limited number of articles. The clinical significance may also be limited due to the prevalence of digestive tract cancers. Lastly, as shown in Table 1, the studies included in the meta-analysis are mostly involving Asian patients, with only two articles from non-Asian ethnicity, therefore, further studies on Caucasian, African and other populations are needed.

In conclusion, despite these limitations, our meta-analysis found out that the expression profile of miRNAs in saliva achieves a relatively high sensitivity and specificity in discriminating cancer patients from healthy subjects. These findings provide important evidence for further development of a salivary miRNA-based noninvasive method for diagnosing cancer in the future. Further large-scale prospective studies are warranted to confirm our analysis.

## Supporting Information

S1 PRISMA Checklist (DOC)

S1 Fig Details of QUADAS-2 quality assessment of each included study (QUADAS-2 tool). (DOCX)

S2 Fig Sensitivity analysis of diagnostic odds ratio (DOR). (DOCX)

S1 Table Detailed information of meta-regression. (DOCX)

S2 Table List of excluded full-text articles. (XLSX)

S1 Text Search strategies. (DOCX)
