# Supplementary figures and images for: The Potential Use of Salivary miRNAs as Promising Biomarkers for Detection of Cancer: A Meta-Analysis
Source: PLoS One. 2016 Nov 10;11(11):e0166303. doi: 10.1371/journal.pone.0166303 (PMC5104484; doi:10.1371/journal.pone.0166303)

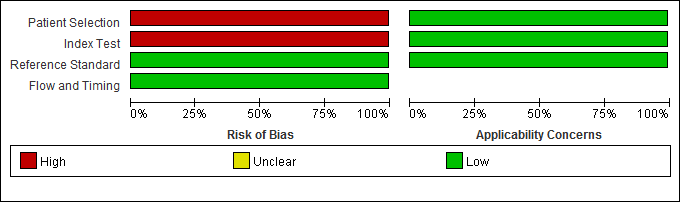


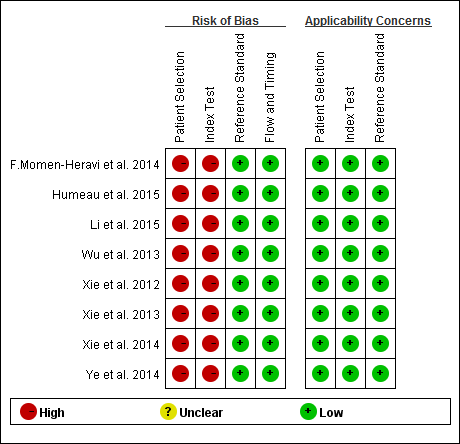


S1 Fig. Details of QUADAS-2 quality assessment of each included study (QUADAS-2 tool).

Supplement: S1 Fig — (DOCX) [file pone.0166303.s002.docx]

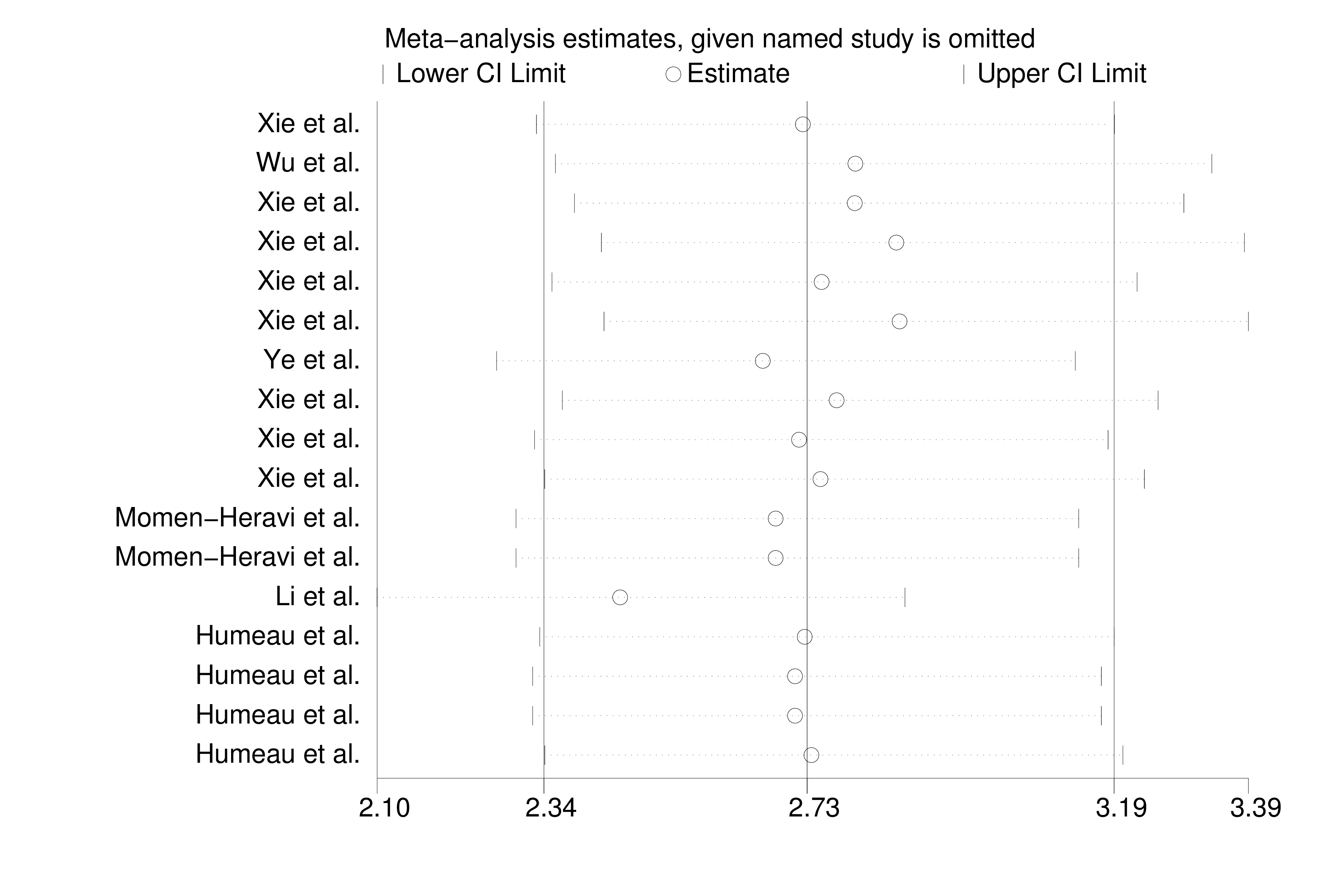


**S2 Fig. Sensitivity analysis of diagnostic odds ratio (DOR)**

Supplement: S2 Fig — (DOCX) [file pone.0166303.s003.docx]
