# Supplementary material for: The Potential Use of Salivary miRNAs as Promising Biomarkers for Detection of Cancer: A Meta-Analysis
Source: PLoS One. 2016 Nov 10;11(11):e0166303. doi: 10.1371/journal.pone.0166303 (PMC5104484; doi:10.1371/journal.pone.0166303)
Supplement: S1 Table — (DOCX) [file pone.0166303.s004.docx]

S1 Table Detail information of meta-regression

| Variables | Coeff. | Std. Err. | P-value | RDOR | 95%Cl |
| --- | --- | --- | --- | --- | --- |
| Ethnicity | -1.424 | 0.7739 | 0.0870 | 0.24 | 0.05-1.27 |
| Sample size | 0.869 | 0.3326 | 0.0205 | 2.38 | 1.17-4.86 |
| Cancer spectrum | 0.399 | 0.3588 | 0.2850 | 1.49 | 0.69-3.22 |
| MiRNAs | -0.025 | 0.4293 | 0.9544 | 0.98 | 0.39-2.45 |

95%CI: 95% confidence interval; Coeff.: Coefficient; Std. Err.: Standard error; RDOR:Relative diagnostic odds ratios.

Ethnicity:Asian/others；Sample size: n≥100 / n<100；Cancer spectrum: esophageal cancer/others；MiRNAs:up-regulated /down-regulated；
