# Supplementary material for: The Potential Use of Salivary miRNAs as Promising Biomarkers for Detection of Cancer: A Meta-Analysis
Source: PLoS One. 2016 Nov 10;11(11):e0166303. doi: 10.1371/journal.pone.0166303 (PMC5104484; doi:10.1371/journal.pone.0166303)
Supplement: S1 Text — (DOCX) [file pone.0166303.s006.docx]

**Literature Search**

**The full details of the databases searched to identify the studies.**

The PUBMED, ISI Web of Science, the Cochrane Library and EMBASE electronic databases were searched using comprehensive search strategies to identify all studies concerning the diagnostic value of saliva miRNAs on cancer detection. All searches were conducted from the earliest date available to October 31, 2015. All indexed journals were included. In order to include all potential studies, we applied various combinations of key words as search items.

**Search Items:** (“Saliva” OR “Spit” OR “Spittle”) AND (“MicroRNAs” OR “miRNAs” OR “miR”) AND (“Cancer” OR “Carcinoma” OR “Tumor”).

Chinese articles were selected by searching WanFang Data and China National knowledge Infrastructure (CNKI) using the same search items. The references of the retrieved articles were manually inspected to find other potential studies. As a search limit, only studies published in English or Chinese were included.
